# Post-transplant trajectories of the CRP-to-albumin ratio identify a late-emerging high-risk phenotype in interstitial lung disease

**DOI:** 10.1186/s12890-026-04648-7

**Published:** 2026-09-01

**Authors:** Ekaterina Krauss, Silke Tello, Luise Wilke, Janine Sommerlad, Gani Oruqaj, Natascha Sommer, Martin Reichert, Andreas Hecker, Anita Windhorst, Matthias Hecker, Andreas Guenther, Sabina A. Guler, Stefan Kuhnert

**Affiliations:** 1 Department of Internal Medicine II, University Hospital Giessen and Marburg, Justus-Liebig-University Giessen, Member of the German Center for Lung Research (DZL), Giessen, 35392 Germany; 2 European IPF/ILD Registry & Biobank (eurIPFreg/bank, eurILDreg/bank), Klinikstrasse 36, Giessen, 35392 Germany; 3 Center for Interstitial and Rare Lung Diseases, University Hospital Giessen and Marburg, Justus-Liebig-University Giessen, Member of the German Center for Lung Research (DZL), Giessen, 35392 Germany; 4 Excellence Cluster Cardiopulmonary System (ECCPS), Justus-Liebig University Giessen and Johann Wolfgang Goethe University Frankfurt, Giessen, 35392 Germany; 5 Department of General, Visceral, Thoracic and Transplant Surgery, Giessen University Hospital, Justus-Liebig-University, Giessen, 35392 Germany; 6 Institute of Medical Informatics, Justus-Liebig University of Giessen, Giessen, 35392 Germany; 7 Agaplesion Lung Clinic “Evangelisches Krankenhaus Mittelhessen”, Giessen, 35398 Germany; 8 Institute for Lung Health, Giessen, 35392 Germany; 9 Department for Pulmonary Medicine, Allergology and Clinical Immunology, Inselspital, Bern University Hospital, University of Bern, Bern, Switzerland; 10 Department for BioMedical Research (DBMR), Lung Precision Medicine (LPM), University of Bern, Bern, Switzerland

**Keywords:** Interstitial lung disease, Lung transplantation, Frailty, CRP-to-albumin ratio, Trajectory analysis, eurILDreg, eurIPFreg

## Abstract

**Background:**

After bilateral lung transplantation for interstitial lung disease (ILD), most recipients recover well, but some deteriorate over the following years. The C-reactive protein–to–albumin ratio (CAR) combines a marker of systemic inflammation (CRP) with one of nutritional and hepatic-synthetic reserve (albumin); a rising ratio reflects worsening inflammation against falling reserve. We assessed whether tracking CAR over time identifies the subgroup at risk.

**Methods:**

We studied 138 consecutive adult ILD recipients of bilateral lung transplants (Giessen, 2000 to 2026), with a median of 13 paired CRP and albumin measurements each. Using group-based trajectory modelling, we grouped patients by the trajectory of their CAR from the first routine follow-up visit (month 4) to year 5, then related the groups to five-year survival and the Clinical Frailty Scale (CFS).

**Results:**

Three CAR trajectories emerged. Most patients were stable-low (39%, CAR near zero throughout) or resolving (38%, initially high then falling). The key group was CAR-rising (23%): at four months these patients were indistinguishable from the best-recovering stable-low group but then climbed steadily over the following years. Five-year survival was 92%, 61% and 69% in the stable-low, rising and resolving groups (log-rank *p* = 0.029; rising versus stable-low hazard ratio 4.5, 95% CI 1.2 to 17.1). A landmark analysis among patients alive at year 2 confirmed this late risk: 33% of the rising group died over the next three years versus 5% of the stable-low group (*p* = 0.005). In the pre-specified model, phenotype membership was not predicted by ILD subtype or LAS at listing (likelihood-ratio *p* = 0.16); pre-transplant CAR itself differed modestly across the groups (*p* = 0.049) but was not prognostic for survival. The CFS trajectory, scored independently of CRP and albumin, showed the same pattern: groups were equally frail before transplant (*p* = 0.83) but separated afterwards (*p* = 0.01 at 4 months, *p* = 0.001 at 5 years), the adverse groups drifting back toward frailty.

**Conclusions:**

A simple test already measured at every post-transplant visit, the CRP-to-albumin ratio, can be read as a trajectory to identify the ILD recipients who enter an inflammatory and nutritional decline associated with worse five-year survival. These findings are hypothesis-generating, and further work is needed to establish what drives the CAR trajectory before any clinical application.

**Trial registration:**

The analysis draws on two registries: the European IPF Registry and Biobank (eurIPFreg; ClinicalTrials.gov NCT02951416, registered 1 November 2016) and the European ILD Registry and Biobank (eurILDreg; German Clinical Trials Register DRKS00028968, registered 26 July 2022).

**Supplementary Information:**

The online version contains supplementary material available at https://doi.org/10.1186/s12890-026-04648-7 .

## Background

Lung transplantation is an effective intervention for end-stage interstitial lung disease (ILD), with most recipients showing substantial functional recovery and reversal of pre-transplant frailty within the first months [1]. A clinically important subgroup, however, deteriorates during long-term follow-up. In the previously published eurILDreg ILD-LTx cohort, some of the recipients did not improve their Clinical Frailty Scale (CFS) [2] status by year 5 post-LTx, and experienced higher mortality compared with patients who maintained the early recovery trajectory [1]. Early identification of patients likely to follow this unfavourable trajectory is crucial; however, effective strategies to do so remain uncertain.

The mechanisms underlying some of the heterogeneity in post-transplant recovery are poorly understood. Inflammation and nutritional depletion have typically been investigated through cross-sectional and short-term studies in critical care and solid organ transplantation populations, limiting the generalisability of single-timepoint biomarker thresholds to long-term post-LTx surveillance [3–6]. The C-reactive protein–to–albumin ratio (CAR) and the modified Glasgow Prognostic Score (mGPS) integrate inflammatory and nutritional features into validated, low-cost prognostic instruments [7–9] that are based on biomarkers measured at every routine clinical visit. A higher CAR denotes greater systemic inflammation (higher CRP) relative to synthetic and nutritional reserve (lower albumin), so that a rising ratio reflects worsening inflammation against falling reserve. Because albumin is a negative acute-phase reactant that also reflects hepatic synthesis and fluid status, the term inflammatory-nutritional is used here as shorthand for this composite signal rather than as a specifically nutritional measure. However, reported optimal cut-offs vary approximately 15-fold across acutely ill populations [3–5], precluding extrapolation to the long-term post-LTx context. Whether inflammatory–nutritional status instead follows distinct longitudinal trajectories in ILD-LTx recipients, and whether such trajectories complement the clinical recovery patterns previously characterised in this cohort [1], has not been addressed.

We hypothesised that longitudinal CAR characterised from month 4 to year 5 post-LTx would reveal distinct trajectory phenotypes in adult ILD bilateral LTx recipients. The specific aims were to (i) identify CAR trajectory phenotypes using group-based trajectory modelling in the pre-specified post-acute analytic window, (ii) examine pre-LTx characteristics associated with phenotype assignment, (iii) characterise correspondence with the CFS trajectory category in our cohort, and (iv) explore the association of phenotypes with all-cause mortality.

## Methods

### Study design and cohort

This is a single-centre, retrospective cohort analysis of consecutive adult patients with ILD undergoing bilateral lung transplantation between December 2000 and February 2026 at the Lung Transplant Centre and the Center for Interstitial and Rare Lung Diseases, University of Giessen and Marburg Lung Center (UGMLC), Giessen site. Patients were enrolled in the European Registry for Idiopathic Pulmonary Fibrosis (IPF, eurIPFreg) or its successor, the European Registry for Interstitial Lung Diseases (eurILDreg), operating under ethical approval from the Ethics Committee of the Justus-Liebig University Giessen (approval no. 111/08). Registration: ClinicalTrials.gov NCT02951416 (eurIPFreg) and DRKS00028968 (eurILDreg). Written informed consent for participation and publication was obtained from all patients [8, 9].

ILD diagnoses were established in accordance with the current ATS/ERS/JRS/ALAT clinical practice guideline through multidisciplinary discussion [10]. Inclusion criteria were age ≥ 18 years and bilateral LTx with ILD as the primary indication. Single-LTx recipients and multi-organ recipients were excluded; a retained native lung is a persistent, asymmetric source of systemic inflammation that would confound a whole-body inflammatory-nutritional ratio, so only bilateral recipients were included. Peri-transplant changes in the CFS and long-term survival in the ILD subcohort from our centre have been reported previously [1], as have longitudinal trajectories of pulmonary function, right heart performance, comorbidity burden and routine laboratory parameters [11].

### Inflammatory–nutritional markers

Serum C-reactive protein (CRP, mg/L) and albumin (g/L) were measured at certified institutional laboratories of the Universities of Giessen and Marburg using standardised assays (ADVIA Chemistry XPT, Siemens, Germany). CRP is a hepatic acute-phase protein synthesised in response to interleukin-6 signalling and reflects the magnitude of systemic inflammation; serum albumin is a negative acute-phase protein that integrates hepatic synthetic function, the magnitude of inflammatory catabolism, and chronic nutritional state. The CRP-to-albumin ratio (CAR) was calculated as CRP (mg/L) ÷ albumin (g/L) and analysed as a continuous variable on the log2 scale [3]. All available paired measurements were retained; infection-associated or otherwise elevated values were not excluded, because the trajectory is intended to integrate the systemic inflammatory–nutritional signal over years, including intercurrent inflammation, and excluding such values would require unblinded adjudication of each measurement and would risk removing the excursions that characterise the adverse phenotypes. The modified Glasgow Prognostic Score (mGPS), a related categorical inflammatory–nutritional index defined by established CRP and albumin cut-offs [7], is referenced for context only and was not modelled in the present analysis.

### Covariates

Pre-LTx covariates included age at LTx, sex, body mass index (BMI), ILD subtype (IPF versus non-IPF progressive pulmonary fibrosis [PPF]), GAP stage at listing, six-minute walk distance (6MWD) at listing, and Lung Allocation Score (LAS) at listing. Peri-operative covariates (intraoperative extracorporeal membrane oxygenation [ECMO], mean ischaemia time, ICU length of stay, and mechanical ventilation duration) were recorded as previously described [1, 11].

### Causal framing and directed acyclic graph

**Fig. 1:**
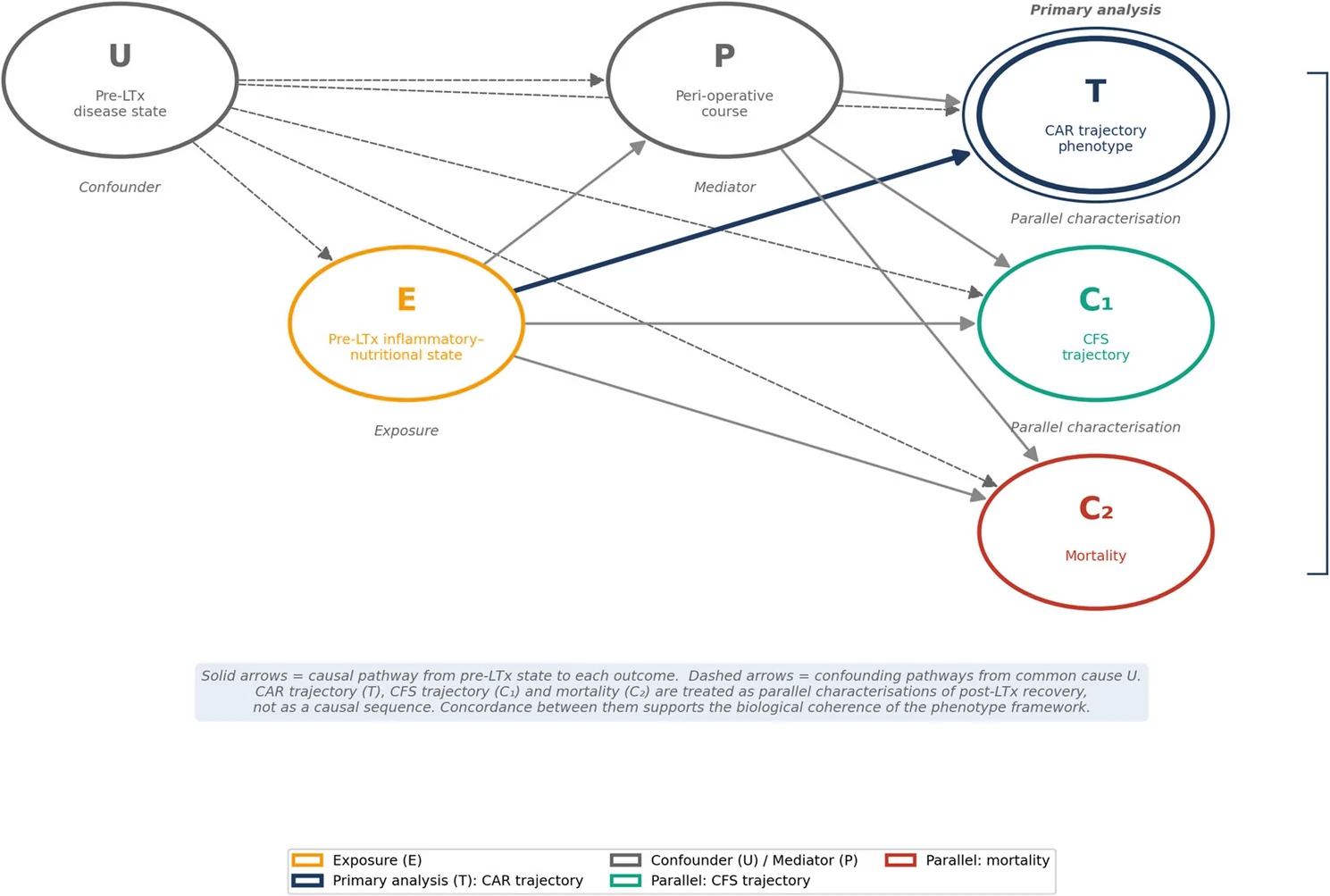
Directed acyclic graph (DAG) framing the analytical strategy. U = pre-LTx disease state (confounder); E = pre-LTx inflammatory–nutritional state (exposure); P = peri-operative course (mediator); T = CAR trajectory phenotype (primary analysis); C_1_ = CFS trajectory category; C_2_ = all-cause mortality. Solid arrows show the causal pathway from pre-LTx state to each outcome. Dashed arrows show confounding pathways from common cause U. T, C₁ and C₂ are treated as three parallel characterisations of post-LTx recovery rather than a causal sequence

To clarify the role of each variable, a directed acyclic graph (DAG) was specified a priori (Fig. 1). The DAG distinguishes pre-LTx disease state as a common cause (confounder) of both pre-LTx inflammatory–nutritional state (the exposure) and the peri-operative course (mediator). The inflammatory–nutritional trajectory phenotype (T), the CFS trajectory category (C_1_), and all-cause mortality (C_2_) are treated as *three parallel characterisations of post-LTx recovery;* the CAR trajectory phenotype is the analysis of primary interest; correspondence with C_1_ and C_2_ supports the face validity (CFS) and predictive validity (mortality) of the phenotype framework.

### Statistical analysis

Inflammatory–nutritional trajectory phenotypes were identified by group-based trajectory modelling (GBTM) [12, 13] applied to log2-transformed CAR within a pre-specified window from month 4 (day 120) to year 5 (day 1825) or event (death, re-transplantation) post-LTx. Quadratic time terms were permitted, and estimation used expectation–maximisation with 25 random initialisations. The number of groups was selected by a minimum-group-size threshold (≥ 5% of the analytic cohort) and clinical interpretability, supported by the Bayesian Information Criterion (BIC); The three-group solution was retained on the basis of classification certainty, parsimony and clinical interpretability, with a mean posterior probability of phenotype assignment of 0.91 (all groups at or above 0.85); formal information criteria for competing numbers of latent classes are not tabulated here. Patients with fewer than three paired CAR measurements within the window were excluded, leaving 104 of 138 as the analytic cohort (34 excluded, 25%). Pre-LTx predictors of phenotype were examined by multinomial logistic regression with two pre-specified variables, ILD subtype (IPF versus non-IPF PPF) [10] and LAS at listing.

Correspondence with the frailty trajectory used the CFS framework [2] with states classified as fit (1 to 3), vulnerable (4), or frail (5 to 9) and post-transplant change as improved, unchanged, or worsened. The CFS score distribution at each timepoint (pre-LTx, 4 months, and 5 years or last contact) was compared across phenotypes by Kruskal-Wallis test to assess convergent validity. All-cause mortality was assessed by Kaplan–Meier with multivariate and Holm-adjusted pairwise log-rank tests, censored at 5 years; hazard ratios relative to the CAR-stable-low phenotype were estimated by Cox regression with the proportional-hazards assumption verified by scaled Schoenfeld residuals, and a landmark analysis among patients alive at year 2 tested for late mortality. Analyses used Python 3.12; two-sided *p* < 0.05 was considered significant.

## Results

### Cohort characteristics and inflammatory–nutritional trajectory phenotypes

Between December 2000 and February 2026, 138 patients with ILD underwent bilateral LTx at our centre and met inclusion criteria (cohort characteristics in Table 1). Median age at LTx was 58.5 years (IQR 52.9–62.6); 63% of the patients were male. The cohort included 47 patients (34%) with IPF and 91 (66%) with non-IPF PPF.

Patient characteristics by phenotype are shown in Table 1. Demographics (age, sex, BMI) and pre-LTx pulmonary function did not differ significantly across phenotypes. IPF was of similar frequency across phenotypes (CAR-stable-low 15 of 41, 37%; CAR-rising 7 of 24, 29%; CAR-resolving 11 of 39, 28%). Phenotype assignment certainty was high: 92 of 104 patients (88%) had a posterior probability of the assigned phenotype exceeding 0.7, and 85 (82%) exceeded 0.8. FVC, DLCO and 6MWD did not differ across CAR phenotypes and showed no rank correlation with phenotype (all *p* ≥ 0.26).

**Table 1:** Cohort characteristics overall and by CAR trajectory phenotype

Variable	Overall (*n* = 138)	CAR-stable-low (*n* = 41)	CAR-rising (*n* = 24)	CAR-resolving (*n* = 39)	*P*
N	138	41	24	39	
*Demographics*					
Age at LTx, years	58.5 [52.9–62.6]	57.2 [52.8–62.6]	59.3 [54.3–62.6]	59.5 [55.2–62.8]	0.430
Male sex, n (%)	86 (63%)	25 (61%)	15 (62%)	24 (62%)	0.993
BMI at LTx, kg/m²	24.8 [21.9–27.1]	24.2 [22.2–27.1]	25.1 [22.8–26.8]	25.0 [21.9–26.9]	0.926
*ILD characteristics*					
IPF, *n* (%)	47 (34%)	15 (37%)	7 (29%)	11 (28%)	0.690
Non-IPF PPF, *n* (%)	91 (66%)	26 (63%)	17 (71%)	28 (72%)	0.690
*Pre-LTx functional status*					
LAS at listing	42.7 [37.0–56.3]	39.0 [36.6–45.9]	42.8 [38.0–57.2]	44.3 [37.7–63.3]	0.209
FVC, % predicted	67.6 [49.4–80.2]	63.5 [46.2–75.2]	74.2 [50.4–85.3]	68.6 [51.8–78.8]	0.517
DLCO, % predicted	54.4 [45.5–63.7]	54.5 [50.2–64.1]	56.1 [48.5–70.0]	54.3 [44.5–62.2]	0.760
6MWD at listing, m	320 [219–450]	315 [198–458]	392 [286–441]	292 [189–330]	0.287
*Baseline inflammatory–nutritional state*					
CRP, mg/L	31.1 [11.5–60.0]	23.8 [10.6–48.3]	55.6 [26.9–120.2]	32.4 [12.8–54.7]	0.058
Albumin, g/L	32.0 [29.0–35.8]	33.1 [29.6–37.0]	30.2 [29.1–36.3]	32.1 [28.8–35.5]	0.465
CAR (CRP/Alb)	0.96 [0.33–1.94]	0.67 [0.31–1.49]	1.83 [0.84–4.28]	1.15 [0.39–1.92]	0.049
*Peri-operative course*					
Ischaemia time, min	358 [323–394]	360 [330–402]	376 [333–408]	344 [310–374]	0.075
ICU LOS, days	20 [10–36]	20 [12–36]	20 [8–30]	14 [10–30]	0.785
Ventilation, hours	53 [18–432]	132 [24–450]	42 [14–216]	34 [13–372]	0.170
Hospital LOS, days	36 [21–60]	38 [25–59]	32 [22–52]	31 [21–60]	0.785
*Outcomes*					
Deceased at follow-up, *n* (%)	66 (48%)	14 (34%)	12 (50%)	18 (46%)	0.380
Follow-up, days	1704 [678–2794]	2460 [1357–3223]	1715 [1444–2442]	1717 [842–2562]	0.137

Values are median [IQR] for continuous variables and *n* (%) for categorical variables. *P*-values compare across the three CAR phenotype groups (Kruskal–Wallis for continuous, chi-square for categorical). The Overall column reflects all 138 bilateral-LTx recipients; the phenotype columns reflect the 104-patient trajectory-analytic subset (those with at least three paired CRP/albumin measurements in the month-4 to year-5 window), so the three phenotype columns do not sum to the Overall column. Deceased at follow-up reflects deaths over the entire observation period and is not censored at five years; it therefore differs from the five-year Kaplan–Meier estimates, particularly for CAR-stable-low, which has the longest follow-up

*CAR*  CRP-to-albumin ratio, *CFS*  Clinical Frailty Scale, *6MWD*  Six-minute walk distance, *LAS*  Lung Allocation Score, *ICU*  Intensive care unit

**Fig. 2:**
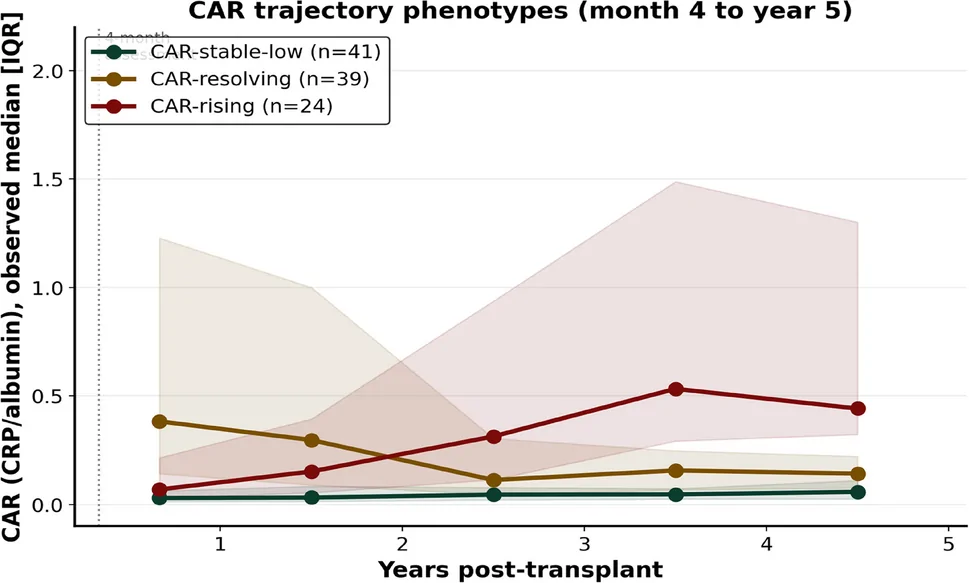
CAR trajectory phenotypes identified by group-based trajectory modelling. Three-group GBTM solution overlaid on individual patient trajectories. Lines represent group-mean trajectories with 95% confidence ribbons; individual patient trajectories shown in faint group colour. CAR-stable-low (green), CAR-rising (red), CAR-resolving (orange). CAR = CRP-to-albumin ratio

A total of 2,285 paired CRP–albumin measurements were available, with a median of 13 per patient (IQR 6–23). Of the 138 patients, 104 (75%) had ≥ 3 paired CRP and albumin measurements within the pre-specified month-4 to year-5 window and constituted the primary analytic cohort for trajectory modelling. The median interval between consecutive paired measurements was 67 days (IQR 29 to 133). The excluded patients differed from the included only in follow-up duration; 18 had no paired measurement at all after month 4 and the remainder only one or two, a minority died before the month-4 window opened (median follow-up among the excluded 0.75 years), and the two groups did not differ in age, sex, disease subtype, body mass index, lung function or eventual mortality (Supplementary Table S1). Group-based trajectory modelling identified three distinct CAR trajectory phenotypes (Fig. 2), here described from the most to the least favourable:

(i) CAR-stable-low (*n* = 41): CAR remained near zero across the full follow-up window, with model-predicted values of 0.03 at month 4 and 0.06 by last visit or year 5.
(ii) CAR-resolving (*n* = 39): CAR was elevated at month 4 (0.50) and declined gradually to 0.13 by last visit or year 5, as patients in this group either recovered or were lost to mortality.
(iii) CAR-rising (*n* = 24): CAR was indistinguishable from CAR-stable-low at month 4 (0.06) but rose progressively over years, with observed median values climbing from 0.09 in the first post-LTx year to 0.53 by year 4 and a model-predicted value of 0.83 by year 5, representing a marked late inflammatory escalation.

### Pre-transplant characteristics associated with phenotype

Multinomial logistic regression was performed in 95 patients with complete data on the two pre-specified pre-LTx variables (ILD subtype, LAS at listing). Full results are presented in Table 2. IPF (versus PPF) showed an OR of 0.72 (95% CI 0.23–2.24, *p* = 0.572) for the CAR-rising phenotype and 0.51 (95% CI 0.18–1.43, *p* = 0.198) for CAR-resolving. LAS at listing showed an OR of 1.51 (95% CI 0.82–2.76, *p* = 0.184) per + 1 SD for CAR-rising and 1.79 (95% CI 1.04–3.06, *p* = 0.034) for CAR-resolving. The overall model was not significant against the intercept-only null (likelihood-ratio *p* = 0.16), indicating that the phenotypes were not jointly distinguished by these two pre-specified variables. Pre-transplant CAR itself was not entered into the model; in univariable comparison it differed modestly across the phenotypes (*p* = 0.049, Table 1), but it was not itself prognostic for survival, so the discriminating prognostic information resides in the post-transplant trajectory rather than in this baseline value.

**Table 2:** Pre-transplant characteristics associated with the CAR trajectory phenotypes (multinomial logistic regression, *n* = 95)

Predictor	CAR-rising vs. CAR-stable-low OR (95% CI), *p*	CAR-resolving vs. CAR-stable-low OR (95% CI), *p*
IPF (vs. non-IPF PPF)	0.72 (0.23–2.24), 0.572	0.51 (0.18–1.43), 0.198
LAS at listing, per + 1 SD	1.51 (0.82–2.76), 0.184	1.79 (1.04–3.06), 0.034
*Model fit*		
Likelihood-ratio test vs. null	χ² = 6.55, df = 4, *p* = 0.161	McFadden pseudo R² = 0.032

Multinomial logistic regression with CAR-stable-low phenotype as reference category

*OR* Odds ratio, *CI* Confidence interval

^*^Statistically significant at *p* < 0.05. ILD subtype was selected as the principal disease-classification axis identified in current ATS/ERS/JRS/ALAT guidance [10]; Baseline CAR was summarised descriptively in Table 1

### Correspondence with the clinical frailty trajectory

We next examined how the CAR trajectory phenotypes related to the peri-transplant frailty trajectory, using the Clinical Frailty Scale (CFS) categories established in our companion analysis [1]. A computable peri-transplant CFS change was defined as the difference between the pre-transplant and four-month scores. CAR and CFS were uncorrelated before transplantation (Spearman rho − 0.03, *p* = 0.76) but became progressively correlated afterwards (rho + 0.28 at four months, *p* = 0.004; +0.39 at five years, *p* = 0.001), so two independently obtained measures that are unrelated at baseline converge over the post-transplant course.

**Fig. 3:**
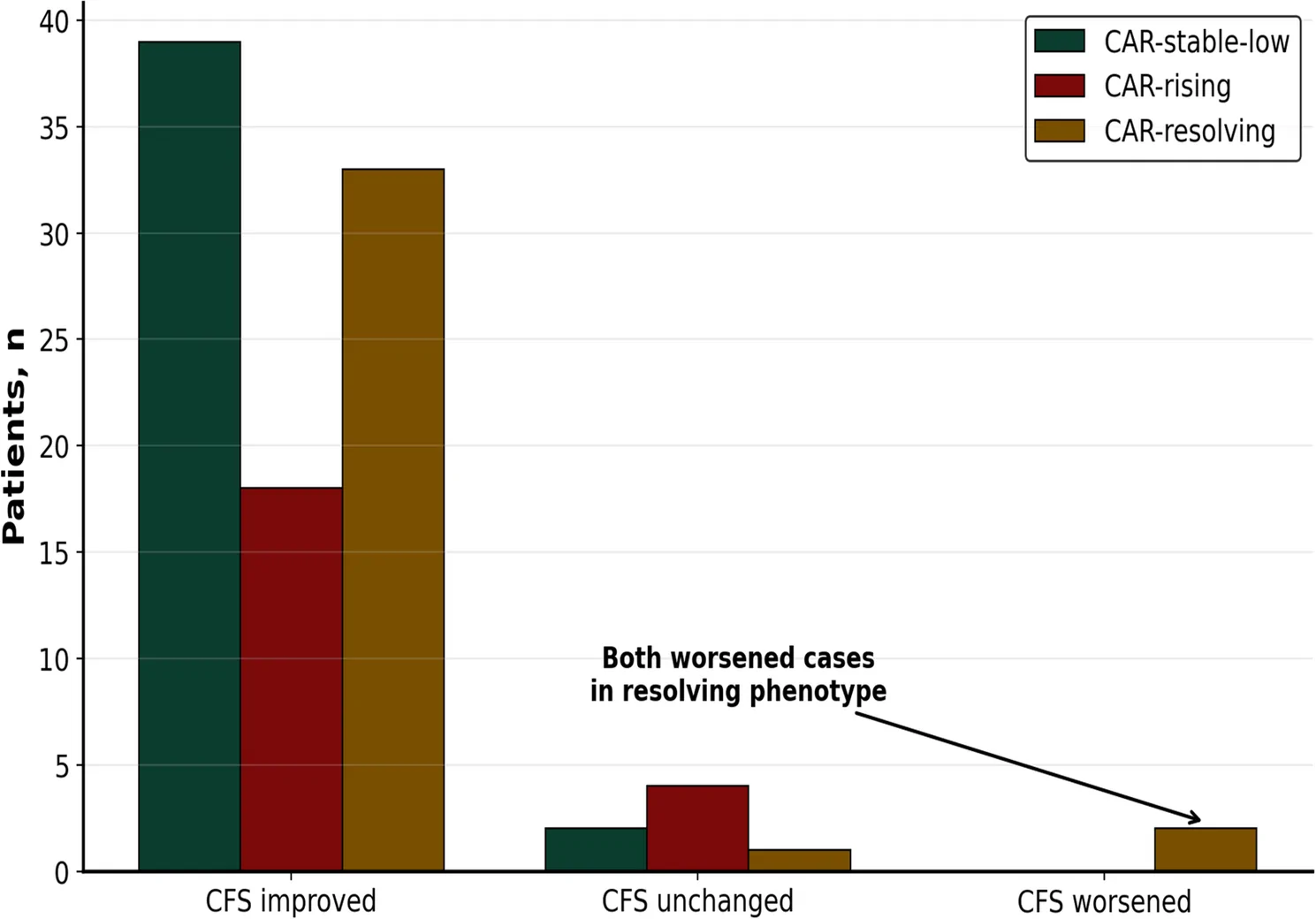
Correspondence of CAR trajectory phenotype with CFS trajectory in our cohort. Grouped bar chart showing the number of patients in each CFS trajectory category (improved, unchanged, worsened) within each CAR trajectory phenotype, in 99 patients of our cohort with both variables available. CFS trajectory categories: ΔCFS ≤ -1 = improved, ΔCFS = 0 = unchanged, ΔCFS ≥ + 1 = worsened

By this categorical measure, frailty improved in the large majority of patients in every phenotype: 95% of CAR-stable-low (39/41), 82% of CAR-rising (18/22) and 92% of CAR-resolving (33/36). The two patients whose frailty worsened both belonged to the CAR-resolving phenotype; none occurred in the other phenotypes (Table 3; Fig. 3). To assess frailty over the full post-transplant period, we compared the distribution of CFS scores across phenotypes at each timepoint.

**Table 3:** Cross-tabulation of CAR trajectory phenotype with CFS trajectory category in our cohort

CFS trajectory category	CAR-stable-low (*n* = 41)	CAR-rising (*n* = 22)	CAR-resolving (*n* = 36)	Overall (*n* = 99)
Improved, *n* (%)	39 (95%)	18 (82%)	33 (92%)	90 (91%)
Unchanged, *n* (%)	2 (5%)	4 (18%)	1 (3%)	7 (7%)
Worsened, *n* (%)	0 (0%)	0 (0%)	2 (6%)	2 (2%)

CFS trajectory categories defined in our cohort following the approach described in [1]. Values are counts (and row percentages). Because CFS worsening was rare (two patients in total), the correspondence is presented descriptively rather than as a formal test of association

*CFS*  Clinical Frailty Scale

### Survival by CAR phenotype

**Fig. 4:**
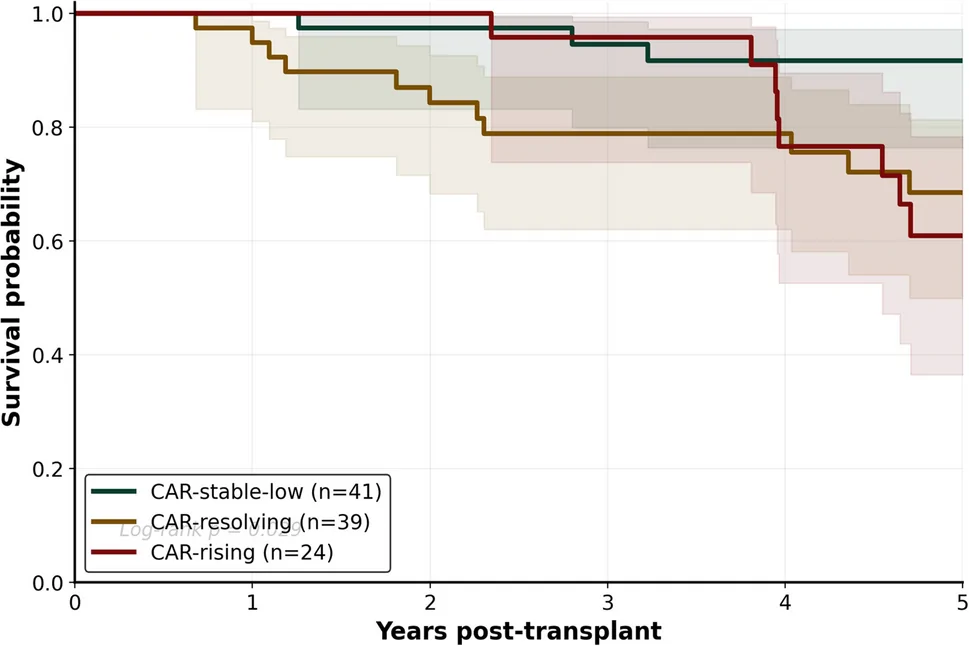
Kaplan–Meier survival curves, stratified by CAR trajectory phenotype. Kaplan–Meier survival curves with 95% confidence intervals, censored at 5 years post-LTx. Numbers at risk are tabulated below the plot. Log-rank test compares all three phenotypes simultaneously

Five-year Kaplan–Meier survival differed across CAR phenotypes (multivariate log-rank χ² = 7.1, df = 2, *p* = 0.029; Fig. 4), at 92% for the CAR-stable-low phenotype, 61% for CAR-rising and 69% for CAR-resolving. Relative to CAR-stable-low, the hazard ratio for all-cause mortality was 4.5 (95% CI 1.2 to 17.1) for CAR-rising and 4.5 (95% CI 1.2 to 16.1) for CAR-resolving, with the proportional-hazards assumption satisfied (scaled Schoenfeld residuals, all *p* > 0.09); the wide confidence intervals reflect the modest number of events. Pre-transplant CAR did not predict survival (Cox hazard ratio per doubling 0.99, 95% CI 0.86 to 1.15, *p* = 0.93; five-year survival 80%, 53% and 74% across baseline CAR tertiles, log-rank *p* = 0.10), indicating that the prognostic signal resides in the post-transplant trajectory rather than the baseline value. Pairwise comparisons (Holm-adjusted log-rank) showed CAR-stable-low survival exceeded both CAR-resolving (*p* = 0.029) and CAR-rising (*p* = 0.041), while the two adverse phenotypes did not differ from one another (*p* = 0.97).

The two adverse phenotypes differed in survival (Fig. 4). In the CAR-resolving phenotype mortality was early, concentrated in the first two post-operative years (2 deaths, 5%, by year 1 and 6, 15%, by year 2). In the CAR-rising phenotype mortality was late: no deaths occurred through year 2 and only one (4%) by year 3, with most events falling between years 3 and 5 (8 deaths, a crude 33% of the phenotype; the Kaplan–Meier five-year estimate is higher because of censoring of patients with shorter follow-up). The CAR-stable-low phenotype had almost no deaths throughout (3 deaths, 7%, over five years). A landmark analysis confined to patients alive at year 2 confirmed the late risk of the CAR-rising phenotype, which remained associated with subsequent death (log-rank *p* = 0.005 versus CAR-stable-low; 33% versus 5% dying between years 2 and 5). These patterns indicate that the two survival deficits arise differently: in CAR-resolving the designation applies only to patients who survived the early peri-operative period and then normalised their inflammatory–nutritional state, a survivor-selection effect, whereas in CAR-rising patients initially indistinguishable from CAR-stable-low accumulate inflammation and deteriorate years later.

**Fig. 5:**
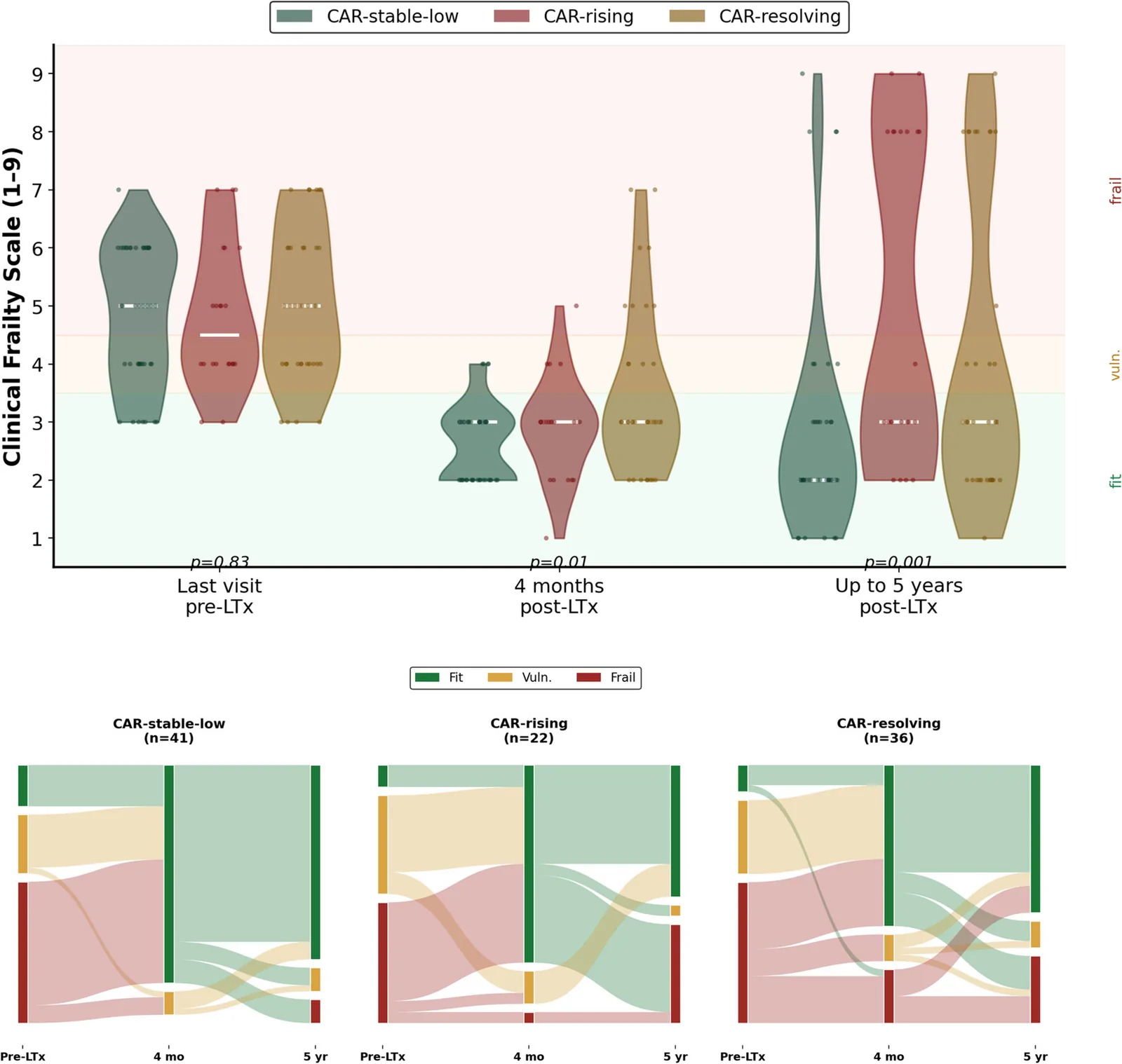
Clinical frailty scale trajectory by CAR phenotype. **a** Violin Plot and (**b**) Sankey diagram show distribution of the Clinical Frailty Scale (CFS, 1 to 9) at the last visit before LTx, at 4 months, and up to 5 years after LTx, shown separately for each CAR trajectory phenotype (violin plots with individual values and medians; shaded bands denote the fit, vulnerable and frail ranges. CFS distributions were indistinguishable across phenotypes before transplantation (Kruskal-Wallis *p* = 0.83) and diverged progressively thereafter (*p* = 0.01 at 4 months; *p* = 0.001 by 5 years), in convergence with the CAR trajectories. CAR = CRP-to-albumin ratio; CFS = Clinical Frailty Scale; LTx = lung transplantation

Of the 138 patients, 131 had a documented CFS (95%) and 104 met the trajectory analytic criteria; 102 satisfied both, and of these 99 had both a pre-transplant and a four-month score and therefore a computable peri-transplant CFS change, the remaining three lacking one of the two scores required to compute it. The CFS distribution was indistinguishable across phenotypes before transplantation (medians 5.0, 4.5 and 5.0 for CAR-stable-low, CAR-rising and CAR-resolving; Kruskal-Wallis p = 0.83) but diverged progressively thereafter, reaching significance at 4 months (all medians 3.0; p = 0.01) and becoming more pronounced by 5 years or last contact (median 2.0 for CAR-stable-low versus 3.0 for both adverse phenotypes; p = 0.001) (Fig. 5). Frailty distributions superimposed at baseline thus separated over the following years, paralleling the CAR trajectories. 

Most recipients of every phenotype were frail or vulnerable before transplantation (frail in 59%, 50% and 58% of the CAR-stable-low, CAR-rising and CAR-resolving phenotypes) and most recovered to fit by 4 months (90%, 82% and 67%). Thereafter the phenotypes diverged: in CAR-stable-low recovery was largely sustained (80% fit, 10% frail at 5 years), whereas both adverse phenotypes showed a partial return toward frailty, the frail proportion rising from 5% at 4 months to 41% at 5 years in CAR-rising and from 22% to 28% in CAR-resolving.

### Sensitivity analyses

Pre-specified sensitivity analyses confirmed the robustness of the three-phenotype solution. A four-group GBTM produced a 3-patient micro-group failing the ≥ 5% minimum-size threshold and was rejected; restriction to patients with ≥ 5 CAR observations in the window reproduced the three-phenotype structure with marginally improved posterior probabilities; and mGPS strata–based phenotyping at fixed timepoints assigned 80% of patients concordantly with the GBTM phenotype dichotomy.

The CAR-rising trajectory reflected observed rather than model-extrapolated escalation. Among the CAR-rising patients, 22 (85%) contributed at least one CAR measurement in years 3–5 and 16 (62%) in years 4–5, with a median last observation at 4.5 years post-LTx. The observed (non-modelled) median CAR rose monotonically across yearly intervals within this phenotype, from 0.09 in the first post-LTx year to 0.18, 0.28 and 0.53 in years 2, 3 and 4 respectively, confirming that the late escalation is present in the raw measurements and is not an artefact of the quadratic time term (supplementary Figure S1). The individual patient trajectories underlying this median are heterogeneous and punctuated by discrete excursions rather than following a uniform climb, so the phenotype captures a tendency toward rising and spiking inflammatory–nutritional signals rather than a smooth monotonic rise in every patient.

## Discussion

This analysis describes three distinct longitudinal trajectories of inflammatory–nutritional status after bilateral lung transplantation for ILD, identified from routinely collected CRP and albumin measurements. Two of the four pre-specified aims yielded the identification of the trajectory phenotypes and their association with mortality; the examination of pre-LTx predictors and the correspondence with the clinical frailty trajectory were supporting analyses shown below. First, we identify a CAR-rising phenotype affecting approximately one-quarter of recipients. These patients are indistinguishable from the favourably-recovering majority at the four-month post-LTx assessment yet undergo a progressive inflammatory–nutritional escalation over the following years. Although this relative escalation is substantial, the absolute CAR values remain low throughout, and the clinical signal therefore lies in the trajectory rather than in any single threshold value. The patients who will eventually deteriorate are not the patients who appear unwell at month 4.

Second, the phenotypes converge with an independently measured axis of post-transplant recovery. When CFS trajectory was examined as a categorical peri-transplant change, the CAR-stable-low phenotype showed clear validity, with 95% of these patients following a favourable CFS trajectory and almost none experiencing peri-operative mortality; the rarity of CFS worsening (two patients) limited what this categorical comparison could show for the other phenotypes. The more informative analysis examined the full CFS distribution at each timepoint. Here the phenotypes were indistinguishable in frailty before transplantation (*p* = 0.83) yet diverged progressively afterwards (*p* = 0.01 at 4 months; *p* = 0.001 by 5 years), the CAR-stable-low phenotype remaining fit while both adverse phenotypes drifted toward frailty. This divergence mirrors the CAR trajectories themselves and provides convergent validity from a second, independent instrument: two distinct measurement systems, a routinely collected laboratory ratio and a CFS, identify the same separation of recovery courses and the same late deterioration. The CAR-resolving phenotype is additionally distinguished by its mortality signal, with an early excess accounting for 6 of its 18 total deaths and 15% of the phenotype dead by year 2, indicating that the trajectory is partly a survivor-selection construct, since only patients who survive the early peri-operative period are observable long enough to demonstrate CAR resolution. Beyond this selection effect, the resolving group’s survival pattern, an early deficit followed by favourable longer-term survival, mirrors its high-then-declining CAR trajectory, and this coherence supports the overarching hypothesis that an elevated CAR marks excess risk that persists until the ratio resolves; the resolving group is therefore positive evidence for the central thesis rather than a peripheral observation. The CAR-rising phenotype, by contrast, is characterised by late deterioration, with no deaths through year 2 but cumulative five-year mortality of 33%. The two adverse phenotypes therefore fail along different timescales, and the convergence of the inflammatory–nutritional and frailty axes strengthens the interpretation that the phenotype structure reflects genuine biological heterogeneity in recovery rather than an artefact of a single marker.

Third, pre-LTx clinical features did not jointly distinguish the phenotypes (likelihood-ratio *p* = 0.16). The trajectory phenotypes therefore capture biological heterogeneity that emerges in the post-LTx period rather than reflecting pre-existing differences identifiable at listing. Phenotype membership was not distinguished by the pre-specified severity variables, and although pre-transplant CAR differed modestly across the groups (*p* = 0.049) it was not itself prognostic; identification of the adverse trajectory therefore requires serial post-transplant measurement rather than any single pre-transplant value.

Earlier work described in narrative form a subgroup of patients who reversed frailty early after LTx but redeveloped frailty in later years [1]. The present analysis provides a quantitative biological correlate of that clinical observation. In the present cohort, IPF was of similar frequency across phenotypes (CAR-stable-low 15 of 41, 37%; CAR-rising 7 of 24, 29%; CAR-resolving 11 of 39, 28%), so disease classification alone did not distinguish the trajectory groups. Recurrence of interstitial disease in the lung allograft has been described [14, 15], but the present data cannot establish whether the CAR-rising trajectory reflects any such host-side biology, immunosuppression-related complications, or subclinical chronic lung allograft dysfunction. Accumulating post-transplant complications and comorbidity are at least as plausible a driver of a rising trajectory as any residual disease biology, and infection, acute rejection and chronic lung allograft dysfunction are common after lung transplantation and are likely contributors. Plausible contributors to a post-transplant rise include cumulative immunosuppression exposure, recurrent or chronic infection, evolving allograft dysfunction and chronic low-grade systemic inflammation; these are candidate drivers to be tested prospectively rather than established mechanisms. Distinguishing these possibilities requires prospective biomarker characterisation including donor-derived cell-free DNA, BAL cytokines, and structured nutritional assessment.

Serial CRP and albumin measurement has three properties that would make it practical to deploy, should the prognostic value of the trajectory phenotypes be confirmed prospectively. Both analytes are measured at routine post-LTx visits in standardised assays at minimal incremental cost. Their derived ratio reflects the integrated inflammatory–nutritional state better than either component alone in fibrotic ILD [11], in critical care [3–5], and in solid organ transplantation [6]. Unlike single-timepoint cut-offs, which vary approximately 15-fold across acutely ill cohorts [3–5], a trajectory-based approach is population-specific and does not require a universal threshold. Whether the same inflammatory–nutritional trajectory is informative beyond ILD is unknown: it may be a general marker of post-transplant recovery applicable across indications, or features specific to ILD biology may make it particularly informative in this group, and validation in broader post-transplant cohorts of other indications is a necessary next step. For the CAR-rising phenotype, these data raise a specific hypothesis for prospective testing, namely that recognising patients whose CAR trajectory diverges from the favourable pattern across the second and third post-transplant year, and evaluating whether closer surveillance alters their course, merits formal study. We emphasise that this is a hypothesis-generating proposal rather than a validated clinical decision rule, given the single-centre, retrospective design and modest subgroup sizes; it requires prospective evaluation before clinical adoption. For CAR-resolving patients, the high early mortality means that intensified peri-operative surveillance captures most of the relevant risk; the potential value of post-transplant trajectory monitoring is concentrated in the CAR-rising subgroup.

Frailty after LTx has been characterised by physical phenotype instruments (Fried, SPPB) and clinical scoring (CFS) in mixed-indication cohorts [16–19], with broadly favourable post-transplant reversal but recognition of a vulnerable subgroup. Biological correlates have typically been investigated through single-timepoint biomarker associations with mortality [3–6] rather than through longitudinal phenotyping. The trajectory-modelling framework, originally developed for chronic disease epidemiology [12, 13], has been applied increasingly to cardiovascular, oncological and respiratory cohorts but rarely to post-transplant biology. The present analysis brings these strands together in an ILD-specific cohort with multi-year follow-up, contributes the CAR-rising phenotype as a previously undescribed subgroup that is clinically silent at the standard four-month assessment, and shows that an inflammatory–nutritional trajectory and an independent frailty trajectory converge on the same recovery phenotypes. Our findings align with the perspective that frailty in fibrotic ILD is a dynamic, multidimensional construct whose biological correlates extend beyond pulmonary function alone [20].

The principal strengths of this analysis are the use of validated inflammatory–nutritional instruments rather than novel composites; a pre-specified analytical plan including DAG-based clarification of variable roles and pre-specified phenotype number and analytical window separating the peri-operative response from the steady-state phenotype; high-density longitudinal CRP/albumin sampling (median 14 paired observations per patient); multi-year follow-up; convergent validity from an independently measured frailty axis [1] that does not share input variables with the CAR; and pre-specified sensitivity analyses demonstrating that the CAR-rising escalation is observed rather than extrapolated.

Several limitations require acknowledgement. In this single-centre retrospective analysis, CRP and albumin were drawn at scheduled clinical visits rather than at protocolised research timepoints. The CAR-resolving phenotype is structurally enriched for early peri-operative survivors: only patients who survive long enough to generate ≥ 3 paired CRP/albumin measurements in the month-4 to year-5 window can be assigned a trajectory, and patients who die in the first months of follow-up cannot be assigned to the resolving phenotype even if their CAR was high at the time of death. This selection mechanism makes the absolute survival values for CAR-resolving an under-estimate of the burden carried by patients who follow this biology, and means the exploratory five-year survival comparison should be read as a description of survival after the early peri-operative period rather than from LTx onward. Follow-up time differs across phenotypes (Table 1: median 2,460 vs. 1,715 vs. 1,717 days for CAR-stable-low, CAR-rising and CAR-resolving; *p* = 0.137). This selection mechanism cannot be fully resolved retrospectively because patients who die within the early peri-operative window do not contribute longitudinal CRP and albumin measurements to the analytic window and therefore cannot be allocated to any trajectory phenotype. A quarter of the cohort was excluded on this basis. Reassuringly, however, the analytic cohort did not differ from the excluded patients in age, sex, disease subtype, body mass index, lung function or eventual mortality (Supplementary Table S1), and the lower rather than higher baseline CAR among the excluded argues against the analytic cohort having been enriched for a more inflamed phenotype. Several clinically relevant exposures and comorbidities that may influence the inflammatory–nutritional trajectory were not retrospectively available for the present analysis, including chronic lung allograft dysfunction (CLAD), bronchiolitis-obliterans syndrome (BOS), cumulative infection burden, acute rejection episodes, donor-specific antibody status, renal function trajectory, post-transplant malignancy, and cumulative immunosuppression exposure. Additional biological dimensions (sarcopenia by imaging, cytokines beyond CRP) were not available. The findings apply to middle-aged bilateral transplant recipients with ILD (median age at transplant 58.5 years, with few recipients above 65) and should not be extrapolated to older recipients, to single-lung recipients, or to non-ILD indications without dedicated validation. Finally, group-based trajectory modelling fits each latent class a smooth polynomial mean trajectory, so a relapsing-remitting pattern defined by recurrent CAR spikes cannot emerge as a distinct class under this specification; identifying such a pattern would require a model of within-person variability, which we propose as future work. The analysis is exploratory and comprises more than thirty reported statistical comparisons, including group differences in baseline characteristics, correlations between CAR and the CFS at three timepoints, and several survival analyses. Apart from the pre-specified pairwise survival comparisons, which were Holm-adjusted, these were not corrected for multiplicity. Given the small size of some subgroups, in particular the 24 patients in the CAR-rising phenotype, and the correspondingly wide confidence intervals, for example a five-year mortality hazard ratio of 4.5 (95% CI 1.2 to 17.1), individual associations should be read as hypothesis-generating and confirmed in an independent cohort before they are relied upon. The number of trajectory groups was a modelling choice supported by classification certainty and clinical interpretability rather than by tabulated information criteria, and it too should be confirmed in an independent cohort. Baseline CAR was not entered into the pre-specified multinomial model of phenotype membership, which included only ILD subtype and LAS at listing; although it differed across the phenotypes in univariable comparison (Table 1), we did not formally test whether baseline CAR independently predicts membership, and such an association cannot be excluded.

Three lines of work follow from these results, all of which are hypothesis-generating. First, whether tracking CAR across routine post-transplant visits identifies at-risk recipients earlier than existing measures is a hypothesis that would need to be tested prospectively before any change to surveillance practice; we do not recommend altering clinical monitoring on the basis of these retrospective data. Second, a protocolised peri-LTx schedule of CRP, albumin and complementary biological markers (donor-derived cell-free DNA, BAL cytokines, structured nutritional assessment) in a multi-centre setting would permit denser trajectory characterisation, enable formal causal mediation analysis, disentangle the CRP-driven and albumin-driven contributions to the rise, and address external validity. Such a schedule should record rather than exclude infection episodes, so that their contribution to the trajectory can be modelled explicitly, and would begin at the month-4 visit, the first stable post-operative assessment after the peri-operative inflammatory surge has settled, continuing at the roughly two-monthly interval observed here. Third, and contingent on first understanding the mechanism linking the trajectory to outcome, an interventional study targeting the CAR-rising phenotype, combining structured nutritional support, individualised rehabilitation and evaluation for occult infection or evolving allograft dysfunction, could test whether trajectory-based stratification translates into modifiable outcomes, with endpoints such as CLAD, BOS and retransplantation. Whether the same approach informs pre-LTx ILD progression and antifibrotic treatment monitoring is a further open question.

## Conclusions

A subgroup of ILD lung transplant recipients enters a slow inflammatory–nutritional decline that the routine four-month assessment cannot detect. Serial CRP and albumin measurements resolve three distinct post-LTx trajectories: a CAR-stable-low majority, a CAR-resolving group whose survival deficit is concentrated in the first two post-operative years, and a previously undescribed CAR-rising phenotype affecting approximately one in four recipients, indistinguishable from the most favourable recoverers at four months yet deteriorating over the following years. Because the discriminating signal resides in the trajectory rather than any single value, plotting each patient’s CAR across routine post-transplant visits, alongside spirometry and the CFS, could flag patients whose ratio rises persistently across the second and third post-LTx year, even from a low absolute value and even when single-timepoint metrics remain reassuring, a possibility that prospective studies should evaluate before any change to clinical surveillance. Convergence with an independent frailty trajectory and with five-year mortality supports the validity of these phenotypes and illustrates how routinely collected biomarkers can be read as trajectories rather than thresholds, and how independent measurement systems can be triangulated to identify subgroups invisible to any single instrument.

## Supplementary Information


Supplementary Material 1. 


## Data Availability

The data that support the findings of this study are not publicly available due to privacy reasons but are available from the corresponding author upon request.
