# Supplementary material for: Post-transplant trajectories of the CRP-to-albumin ratio identify a late-emerging high-risk phenotype in interstitial lung disease
Source: BMC Pulm Med. 2026 Sep 1;26:390. doi: 10.1186/s12890-026-04648-7 (PMC13531901; doi:10.1186/s12890-026-04648-7)
Supplement: Supplementary file 1 [file 12890_2026_4648_MOESM1_ESM.docx]

**Supplementary material**

**Figure S1. Observed CAR values in the CAR-rising phenotype.**

Faint red lines show individual patient CAR measurements; the heavy line shows the observed median with interquartile range within yearly intervals. The monotonic rise across years confirms that the late escalation is present in the raw measurements and is not an artefact of the modelled quadratic time term. CAR = CRP-to-albumin ratio.

**Supplementary Table S1. Comparison of included (analytic cohort) and excluded patients.**

| **Characteristic** | **Included (n=104)** | **Excluded (n=34)** | **p** |
| --- | --- | --- | --- |
| Age at LTx, years | 58.6 [53.7–62.5] | 58.0 [49.9–62.9] | 0.29 |
| Male sex, n (%) | 64 (62) | 22 (65) | 0.84 |
| IPF, n (%) | 31 (30) | 16 (47) | 0.09 |
| BMI, kg/m² | 24.9 [22.3–27.1] | 24.1 [21.0–27.2] | 0.44 |
| LAS at listing | 42.2 [36.8–53.9] | 45.4 [40.3–57.5] | 0.28 |
| FVC, % predicted | 67.0 [49.4–80.3] | 68.2 [48.6–75.5] | 0.68 |
| Baseline CAR | 1.2 [0.4–2.2] | 0.5 [0.2–1.4] | 0.002 |
| Deceased (any follow-up), n (%) | 50 (48) | 16 (47) | 1.00 |
| Follow-up, days | 2053 [1383–3225] | 273 [93–514] | <0.001 |

Values are median [IQR] for continuous variables and n (%) for categorical variables. P-values from the Mann–Whitney U test (continuous) or Fisher's exact test (categorical). LAS, Lung Allocation Score; CAR, CRP-to-albumin ratio; FVC, forced vital capacity; BMI, body mass index.
